# Domiciliary dentistry clinics: a multiple case study in the province of Quebec, Canada

**DOI:** 10.1186/s12913-021-06788-4

**Published:** 2021-09-15

**Authors:** N. Makansi, J. Rousseau, C. Bedos, ACE-Dent Research Group, Linda Gauthier, Laurent Morissette, Isabelle Ducharme, Claire Savage, Shahrokh Esfandiari, Richard Hovey, Mary Ellen Macdonald, Belinda Nicolau, Martine Lévesque, Kim Farrell, Tammy Thomson, Nareg Apelian, Mario Brondani, Félix Girard, Aimée Dawson, Jean-Noel Vergnes, Alessandra Blaizot

**Affiliations:** 1 Faculty of Dentistry, McGill University, 2001 McGill College Ave, Montreal, Quebec H3A 1G1 Canada; 2 School of Rehabilitation, Université de Montréal, 7077 avenue du Parc. Mailing address : C.P. 6128 Centre-ville, Montreal, Quebec H3C 3J7 Canada; 3 Research Center, Institut Universitaire de Gériatrie de Montréal, Montreal, Canada

**Keywords:** Mobile Dentistry, Geriatric Dentistry, Disability, Oral Healthcare, Accessibility, Qualitative research, Case Study

## Abstract

**Background:**

The demand for more flexible and person-centered models of oral healthcare delivery is increasing and while mobile and domiciliary dental services have the potential to increase access to oral healthcare among dependent elderly and people with disabilities; the uptake of this service model by dentists remains low. Therefore, the aim of this study was to understand how existing domiciliary dental services operate within a particular context.

**Methods:**

We used a qualitative descriptive multiple case study design. We studied three independent domiciliary dentistry clinics in the province of Quebec, Canada. We completed observations of 27 domiciliary visits, four of which were in private homes and the remaining 23 in LTCFs. We also conducted semi-structured interviews with dental professionals, patients, and caregivers. We performed a qualitative content analysis using a deductive/inductive coding framework.

**Results:**

We presented a detailed description of the physical and service features of the studied cases. Physical features included the set-up of the mobile clinics, the portable equipment used, and the domiciliary locations of visits. For service features, we described the roles, attitudes, and interactions among those involved on both the providers’ and recipients’ sides, as well as, the logistical and financial aspect of the domiciliary dental services.

**Conclusions:**

Despite variations in setup and years of practice, the three mobile clinics had similar physical and service features. They also faced common logistic challenges but were able to provide services and respond to the high demand for domiciliary dental services. Additional research in different contexts would further contribute to building evidence-based models to help increase the uptake of this type of practice by current and future dental professionals.

**Supplementary Information:**

The online version contains supplementary material available at 10.1186/s12913-021-06788-4.

## Background

Ageing increases the risk of physical, cognitive, and functional decline, which in turn poses challenges in accessing conventional oral healthcare systems (i.e. fixed dental clinics). In western societies, the elderly population is growing and what used to be coined as the population “pyramid” is rapidly broadening at the top tier [1]. Also, more people are keeping their natural teeth into old age, which is increasing the demand for oral healthcare.

In response to the health-related challenges faced by aging populations, the WHO developed an action plan and strategy on ageing. This action plan prepares for a “decade of concrete global action (2020-2030)”, which the WHO had declared as the decade of *Healthy Ageing*. It includes ten priorities for action, among which one is to align health systems to the needs of older people where “older adults get the health care they need -where and when they need it” [2]. This priority for action is particularly relevant in dentistry because, for dependent elderly living at home or in long-term care facilities (LTCFs), accessing traditional dental clinics is challenging and sometimes impossible.

One way to respond to this problem, especially for the dependent elderly, is to support the practice of mobile dental services. This idea is not new: back in 2001, Lee and Thomas wrote that “as our population ages further, portable and mobile dentistry will be a necessity, not a luxury” [3]. It is important to mention that mobile dentistry is an umbrella term for different mobile systems such as equipped vans or domiciliary (also called “portable”) dental services. More specifically, domiciliary dentistry is “a service that reaches out to care for those who cannot reach a service themselves. (It) is intended to include oral healthcare and dental treatment carried out in an environment where the patient is resident either permanently or temporarily, as opposed to that care which is delivered in dental clinics or mobile units (vans). It will normally include residential units and nursing homes, hospitals, day centres and patients’ own homes” [4].

Unfortunately, 20 years after Lee and Thomas’s call, domiciliary dentistry is still in low supply as only a small number of clinicians are leading this kind of practice. The literature cites dental professionals' negative attitudes towards mobile services as an obstacle that impedes their adoption of domiciliary dentistry [5, 6]. Such negative attitudes may be due to logistic and financial concerns but also a lack of training and the apprehension of serving people with complex medical conditions [7]. Moreover, we lack models of practice for domiciliary dentistry that could encourage dentists to adopt this approach and guide them in its implementation.

With evolving demographics, we need to propose alternative models of oral health care services. This is one of the goals of our research program named ACE-Dent (Accessible Clinics and Equity in Dentistry) [8]. More specifically, the aim of this study was to understand how existing domiciliary dental services operate within a particular context in order to inform the development of practice models and provide evidence-based recommendations.

## Methods

### Research design

We conducted a qualitative descriptive multiple case study, which Yin (2014) described as the investigation of contemporary phenomena in real contexts [9]. In our research, we were interested in domiciliary dentistry in the context of private dental care in the Canadian province of Quebec. Quebec is the second most populated province in Canada with a population of approximately 8.5million. In 2020, the percentage of 65 years and over in Quebec was approximately 20% of the total population [10].

We defined a “case” as any mobile dental clinic providing domiciliary dental services. Referring to the Model of Competence [11]; a conceptual framework on person-environment interaction, each case was bounded by human and nonhuman (physical) elements including the dentist(s) and other members of the dental team (i.e. dental assistants and administrative staff), as well as the equipment and the environment in which the dental services were provided.

### Ethics

We obtained human research ethics approval from the Institutional Review Board at McGill University, Montreal, Canada (IRB Study Number A06-E50-18B). Informed consent was obtained from all participants and all methods were carried out in accordance with relevant guidelines and regulations.

### Data collection

We first identified 2 cases (mobile clinics) in the province of Quebec. We conducted non-participant observation and semi-structured interviews with the dental professionals, and when feasible, with patients or their caregivers. Observations were recorded in the form of hand-written field notes and interviews were audio-recorded for analysis purposes. We then identified a third case. However, this came during the COVID 19 pandemic; therefore, data collection for this case was solely through online interviewing.

#### Observations

We completed observations of 27 domiciliary visits, four of which were in private homes and the remaining 23 in LTCFs. The non-participant observations consisted of a member of the research team accompanying the mobile dentist/dental team on select days and observing multiple visits in LTCFs or private homes. The observations took place over ten non-consecutive days. We observed the process of transporting equipment, setting up in a domicile, delivering care, and transitioning between appointments. Before entering a domicile, particularly private homes, the dentist would inform the patient or a family member about the research and ask for their permission to allow the researcher into their home/room. After that, the researcher sought their formal consent.

#### Interviews

We used a combination of semi-structured interviews and informal discussions with five dentists (three owners and two associates), one dental assistant, and two administrators. Interviews with the dental professionals took place whenever it was convenient for the participants to chat; between appointments, during breaks/lunch, in the car while commuting to see patients, and over the phone or videocalls (outside working hours). Interviews with patients or caregivers took place at the domicile, right before or after they interacted with the dentist. When patients were cognitively unable to participate in interviews, we asked to interview the caregiver. In total, we formally interviewed four caregivers and two patients. We used two semi-structured interview guides: one for the dental team and another for the patient/caregiver (see Additional file 1). For the dental teams, we used open ended questions to elicit an in-depth description of the features of domiciliary dentistry including the physical environment, the delivery of care, and interactions with patients and other team members. For patients, the interview guides included questions about their health conditions, why and how they sought domiciliary dental care, and what the experience of domiciliary dentistry meant for them.

### Data analysis

We conducted a qualitative content analysis of the field notes and transcribed interviews. We developed and refined a coding frame to represent the analytical framework (Table 1). The content analysis followed a combination of deductive and inductive coding. The deductive categories (i.e. the distinction between physical and service features) were guided by Rousseau et al.’s Model of Competence [11]. We defined “features” as the characteristics and presentation of the physical and human environments of the bounded cases. NM followed an iterative process of immersion in the text, coding, and theme extraction. This process was supplemented with regular discussions and consultations with other research team members.

**Table 1:** Coding frame for data analysis

Features of Domiciliary Dentistry (DD)		
Categories	Themes	Codes
Physical Features	Domiciliary Dentistry Setup	- The “dental office”
		- Equipment
		- Domiciles (Homes or LTCFs)
Service Features	Main Actors’ Attitudes and Interactions	- Attitudes of service providers
		- Attitudes of patients and caregivers
		- Interactions: dental team, patients, and others involved
	Logistics and Treatment	- Scheduling and planning visits
		- Accessing the domicile
		- Setup and treatment
		- Financial aspects

## Results

We studied three independent cases that we will refer to as clinics A, B, and C. Table 2 provides a comparative description of these clinics. The clinics served dependent elderly and people with disabilities or debilitating health conditions who faced challenges in accessing traditional dental clinics. Home visits were also provided to patients with major depression or agoraphobia.

**Table 2:** Description of cases

Characteristics	Clinic-A	Clinic-B	Clinic-C
Years in operation (at time of study)	30 years	1.5 years	5 years
Mobile team members (on-site)	Dentist (owner) Five associate dentists (part-time basis) Two dental assistants (rotating between associate dentists)	Dentist (owner) Dental assistant	Dentist (owner) Dental assistant Secretary
Remote team members	Two administrative assistants (full-time)	Secretary (full-time)	Administrative assistant (part-time)
Equipment	One portable dental unit with stacked storage case (custom-made) One backpack	One portable dental unit (purchased commercially) One rolling suitcase + backpack Laptop computer	Two portable dental units with stacked storage cases (custom-made) One rolling suitcase Laptop computer Defibrillator
Schedule of service delivery	Owner: 4 days per week Associate dentists: 2-3 days per week	5 days per week	4 days per week
Domiciliary service locations	LTCFs (mainly public) and some private homes	Mainly private homes and some private LTCFs	LTCFs (mainly private)
Dental services provided	Preventive services (i.e. examination and cleaning) Dental fillings Tooth extraction Denture repair Scaling and abscess drainage	Preventive services (i.e. examination and cleaning) Dental fillings Tooth extraction Denture repair Scaling and abscess drainage	Preventive services (i.e. examination and cleaning) Dental fillings Tooth extraction Denture repair Scaling and abscess drainage
Fees	Follows syndicate fee guide (Does not charge a fee for displacement)	Follows syndicate fee guide + Flat displacement fee per patient	Follows syndicate fee guide + Flat displacement fee per patient

The history and organization of the clinics varied. In clinic A, the dentist started offering domiciliary services immediately after graduating from dental school and gradually grew the practice bringing associate dentists, dental assistants, and clinic managers into the team. While the dental assistants rotated between associate dentists, the owner and one of the associates provided services independently, without an assistant. The two full-time administrators (clinic manager and assistant-manager) managed the scheduling, billing, and coordination of appointments while remotely monitoring daily developments.

In the other two clinics (B and C), the dentists had transitioned to domiciliary dentistry after a few years of practicing in traditional clinics. They launched their services in teams of two, working closely with their dental assistants. One of them later hired a secretary who traveled with the dentist and the dental assistant in order to complete patients’ files on-site. This, according to the dentist, saved time and allowed them to see more patients per day.

In the next sections, we will describe the physical and service features of the clinics we studied (Table 1). In terms of the physical environment, we will describe what constitutes the “dental office” in domiciliary dentistry; the equipment used; and the domiciliary settings where service is provided. In terms of service features, we will describe the main actors; their interactions; and the logistic and financial aspects of domiciliary services.

### Physical features

#### Domiciliary dentistry setup

##### The “Dental office”

There were three spaces that constituted the physical environment of the mobile clinics: a garage, the patient’s domicile, and the workplace of administrative staff (not observed). Each clinic had a dedicated garage space for equipment storage and sterilization (in dentists’ own-homes or a rented garage space); visits were carried out in the patients’ homes or in LTCFs; and clinics’ administrative staff used their own homes as their workplace (except one secretary that traveled with the dental team).

##### Equipment

**Fig. 1:**
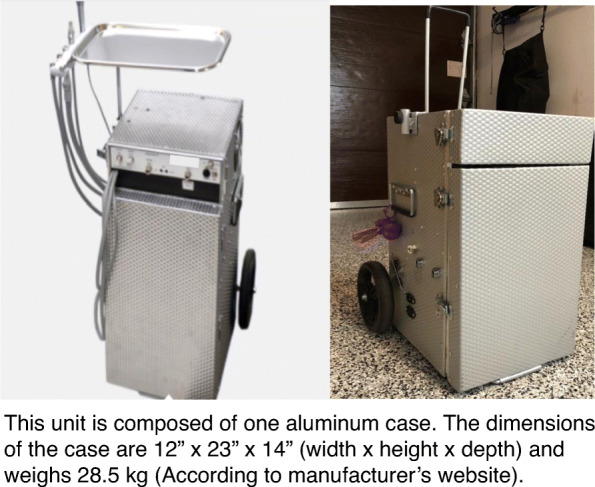
Portable dental unit- commercial model

**Fig. 2:**
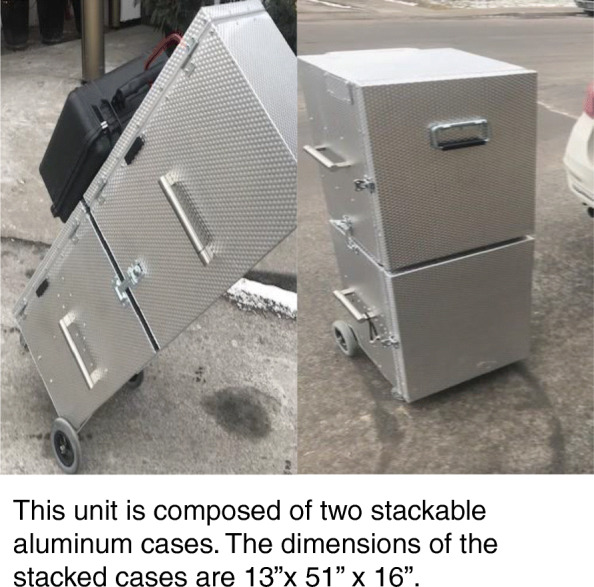
Custom-made portable dental unit

We observed two types of portable dental units: Clinic B used a commercial model (Fig. 1, 1A) which was retailing at approximately CAD 8,000. The other two clinics used custom-made dental units (Fig. 2). The owner of Clinic A had envisioned the design based on personal experience and collaborated with a technician to execute it. According to this dentist, the unit cost around CAD10,000. It consisted of two stackable cases: one for the compressor, motor, and water tanks (Fig. 2A), and another case fitted with drawers for storage (Fig. 2B). The mobile dentists used the same instruments and materials typically found in a conventional dental clinic setting. The only exception was x-ray devices; which were prohibited by the Quebec government for use in domiciliary settings

**Fig. 3:**
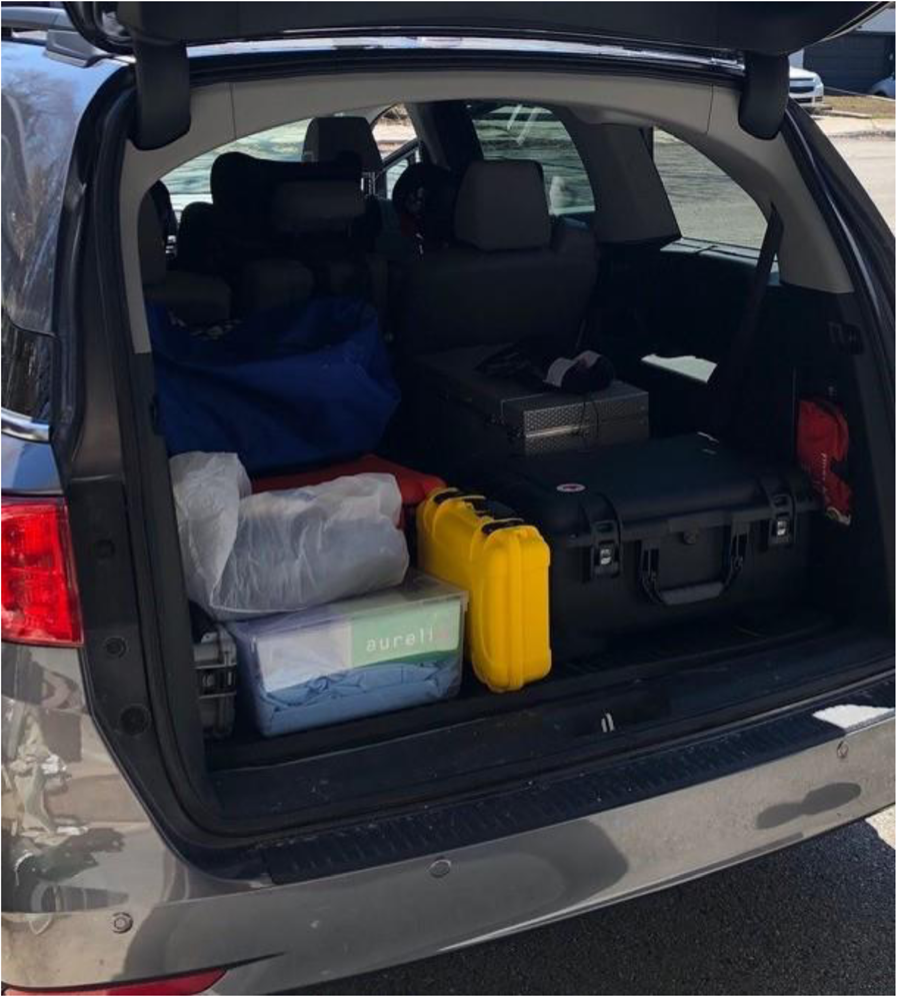
Vehicle with dental equipment for domiciliary visits

**Fig. 4:**
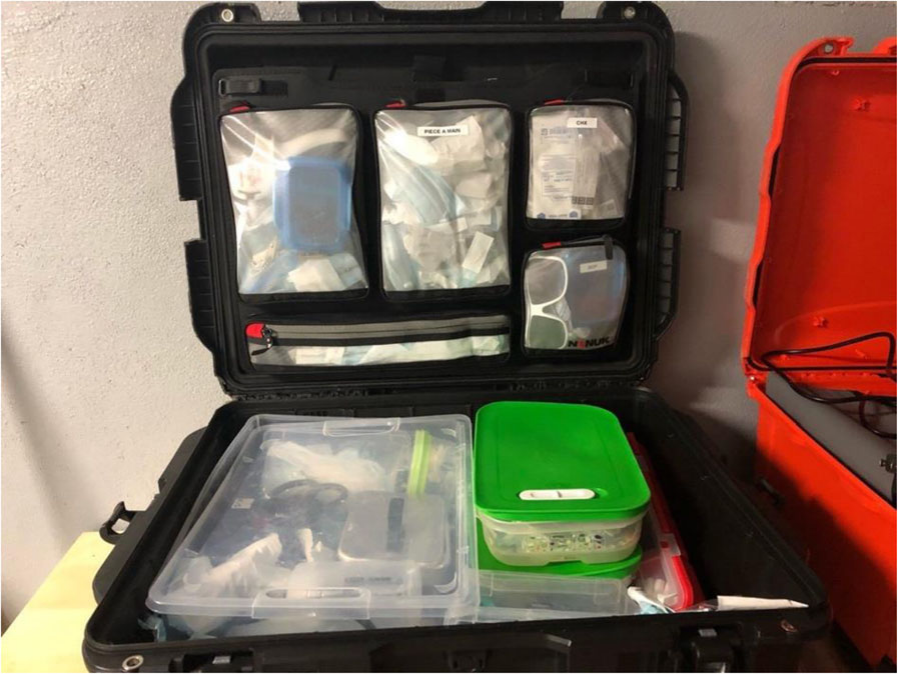
Instruments and equipment packed for domiciliary visits

Clinic C had purchased two sets of the customized units because the dentist wanted to accelerate transitions between patients in LTCFs. In this clinic, while the dentist was finishing up with one patient, the assistant would begin setting up the second protable unit with the next patient. Each workday, the mobile dentists or dental assistants packed their instruments and equipment into their vehicles (Fig. 3). All three clinics typically transported: the dental unit, individual sterilized dental examination kits, dental instruments and material (e.g. impression trays, extraction forceps, dental filling material) and disposables like gloves and bibs (Fig. 4).

**Fig. 5:**
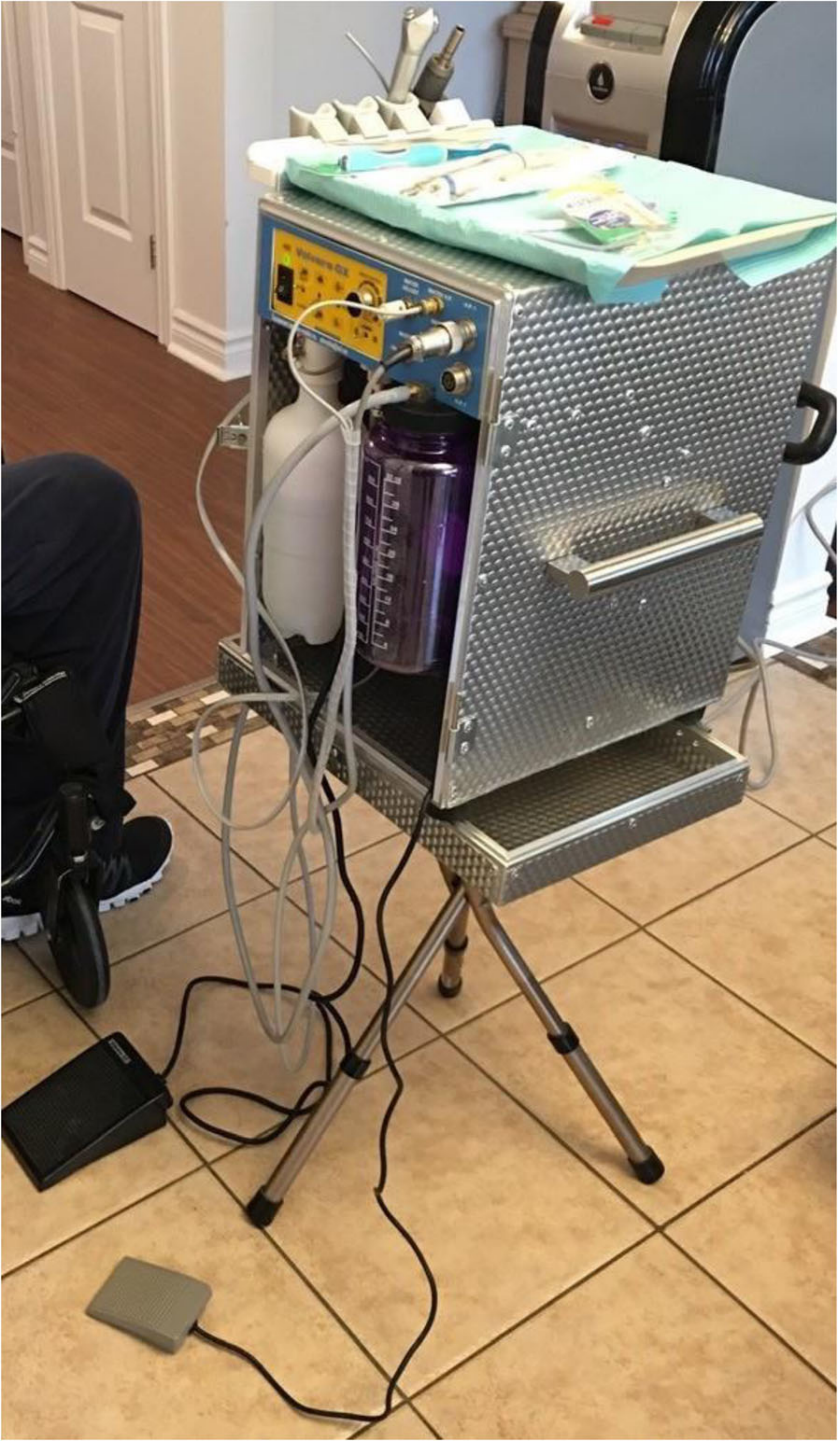
Compact custom-made portable unit for home visits

During this research, the same dentist who designed the customized stackable units was finalizing a new design of a compact unit for homes (Fig. 5). The new unit was lighter and easier to carry, the dentist explained:

*The [smaller]unit is built with an electric motor with variable speed controlled by electric pedal, and a small compressor for air and water. No air reservoir is needed.*

##### Domiciles

The three clinics had different profiles, clinic B primarily offered home visits, clinic C focused on LTCFs, and clinic A served a mix of homes and LTCFs with a focus on the latter. Some dentists expressed a preference for working in LTCFs and described them as more “efficient” due to the hospital-like set up and the support of nursing staff; the potential to see multiple patients in one trip; and avoidance of moving equipment in, out, and between homes. According to those dentists, the number of patients they visited per day ranged from seven to ten. On the other hand, the dentist who focused on home-visits was able to see four to five patients per day:

*The maximum I see is four patients* (per day)*, five I would be exhausted. I must reserve 1.5-2 hours per patient given the commute, set up, etc... So, if we calculate all that, the maximum number of patients I can have in my practice is 300. It's not a lot. I used to see up to 12 patients before (in a traditional dental office)*. (Dentist, Clinic B)

### Service features

#### Main actors’ attitudes and interactions

##### Attitudes of service providers

The dentists expressed different motivations for starting their mobile practice, their personal stories constituting a mix of altruism and self-interest. First, they described a desire to give and help others that was fueled by personal life events: one dentist was motivated by their work with geriatric patients before making the switch to mobile dentistry; another described having a “transformational” personal experience as a caregiver; and a third was inspired by a role model in the field. For these dentists driven by altruism, domiciliary dentistry was particularly appealing as it responded to the needs of people that they perceived as vulnerable and underserved. One dentist, for instance, highlighted the importance of helping seniors maintain their autonomy and reside in their homes as late as possible. Referring to the COVID pandemic and its concentration in LTCFs, this dentist emphasized the importance of home-based domiciliary dentistry.

Besides altruism, some dentists highlighted the advantages of having flexible working hours and a profitable model of practice. One of them, for instance, explained that the switch from a traditional to a mobile practice improved their work-family life balance and eliminated the high running-expenses of a fixed clinic.

*I wanted a family and easier life, so it was easier knowing we don't have to work in the evening. If the baby is sick or something, it’s not a big structure to keep running.* (Dentist, Clinic C)

The mobile dental teams shared some notable personality traits including patience, adaptability, and resilience that seemed essential for this type of practice. Patience was particularly needed when dentists were starting their mobile clinics. They described the process as a “big learning curve” due to the lack of resources for mobile clinics compared to traditional clinics (such as guidelines, equipment, and training). For this reason, they acknowledged, some dentists may quickly give up on the idea of domiciliary services.

Dentists also needed to be patient and adaptable on a daily basis as they navigated through domiciliary settings and dealt with various challenges, such as poorly accessible buildings, uncooperative patients, or other external variables like bad weather and lack of parking space (more details on these issues in the following sections). The administrative dental staff also needed patience and good organizing skills as they coordinated the logistics, appointments, and payments with patients or their caregivers, including family members or the LTCF staff. Adaptability and persistence were such prominent and somehow unusual qualities for dental professionals that one dentist concluded: “this type of practice is not for everyone!”. (Associate Dentist, Clinic A)

Our data also showed that the dentists and their staff were able to develop their skills over the years and experience positive impacts on their personal lives. Although emotionally demanding at first, the participants explained that with time, working with vulnerable patients made them more resilient and improved their ability to manage their emotions and handle difficult situations. One dental assistant even described the work in domiciliary dentistry as “life-changing”:

*We all will grow old and get to this stage, we will be like them. This made me re-evaluate my own life. I didn’t want to be in a bad relationship…we need to be happy now and live in the moment* (Dental assistant, Clinic C)

#### Attitudes of patients and caregivers

Patients and their caregivers expressed gratitude towards the dentists for offering domiciliary services. Having a mobile dentist was described by one caregiver as “marvelous! Just marvelous!”. A grateful 79-year-old patient diagnosed with Parkinson’s and arthritis described how quickly his physical condition deteriorated forcing him to switch from cane to walker to wheelchair over a short period of time:

*I woke up one morning two years ago and was unable to walk. They took me to the hospital and from there to the nursing home. I had been very regular with my dental visits for over 20 years, so I was delighted to find* [the mobile dentist]! *He is very professional. I feel comfortable with him and trust his opinions.*

Furthermore, participants highlighted human qualities and competence of the dental team describing them as “kind” and “caring” and admiring their preparedness in terms of skills and equipment. According to patients and caregivers, domiciliary dentistry eliminated the challenges they faced when seeking oral healthcare such as difficulties in “travelling” to a clinic; the physical barriers of inaccessible dental offices, and the lack of skills and negative attitudes of some dental professionals.

Getting to a dental clinic was one of the main reported challenges. According to one patient, the commute was “painful” because of old age, poor health condition, and fear of travelling. Additionally, unanticipated delays or no-shows of adapted transport may occur, leading to missed or cancelled appointments. Caregivers also described travelling as complicated, time consuming, and physically demanding for them too; since some caregivers were older adults themselves with varying physical abilities.

*I took her in her wheelchair to the dentist…That was harder on me than her because I have a sore shoulder. I had to put her into the car and then the* (wheel)*chair, and I’m not able to that on my own. I actually adapted but it is just more physically demanding…*(and) *it’s definitely a longer process.* (daughter of elderly patient)

Domiciliary dentistry also eliminated the challenge of accessing poorly adapted clinics and receiving quality dental care. One caregiver, for instance, highlighted the difficulties she used to experience when trying to maneuver her mother’s wheelchair in the small rooms of the dental clinic and how overwhelming this task was. A bed-ridden patient deplored dentists' lack of competence and pointed out that it was hard to find “doctors who [were] specialized [in reference to her disability]”; she also believed that some dentists had negative attitudes and “[did] not want to treat elderly patients”.

#### Interactions between the dental team, patients, and others involved

Communication had several layers that were more complex than in traditional clinical settings and sometimes created challenges. In terms of interactions with patients, one dentist explained that Alzheimer’s was “particularly challenging” for the dental team, as the patient may not recognize the dentist, even after several encounters, making it difficult to establish communication and trust.

Also, it was often necessary for the dentists to involve family members or caregivers due to inability of some patients to consent to treatment. The legal representative or caregiver would give the approval to proceed with a treatment plan and guarantee payment. One dentist describes how they sometimes had to take intra-oral photographs as “evidence” of the work when interacting with skeptical family members.

Moreover, when patients resided in a LTCF, the dental team also communicated with the LTCFs nurses, attendants, and administrative staff to obtain information about the medical history and the list of medications or to give specific instructions pre/post dental treatment. Table 3 presents two vignettes (created from our observation fieldnotes) that illustrate the particularities of communication in domiciliary dentistry.

**Table 3:** Vignettes (based on observation data)

Vignette 1	
The dentist had a follow-up appointment with a 94-year-old patient with Alzheimer’s who resided in a LTCF. From previous encounters, the dentist was aware of the patient’s tendency for aggressive behavior (ex. biting). The patient’s daughter booked the appointment directly with the mobile clinic’s manager; the latter then called the residence staff to relay the dentist’s instruction to give the patient a small dose of tranquilizer prior to the scheduled visit^a^. The patient’s daughter was not present at this appointment. Under medication, the patient was unable to engage verbally with the dentist and appeared in a light sleep. The dentist used non-verbal sensory stimulation to communicate certain commands (for example, applying slight pressure behind the corners of the lips to ask her to open the mouth). After completing the appointment, the dentist called the patient’s daughter to give her an update. He also wrote an update in the patient’s file at the center and then sent an electronic update to his clinic’s manager.	
Vignette 2	
Another patient in a LTCF was refusing to interact with the dental team. She turned her head away every time the dentist tried to approach and explain that the appointment was planned by her son. “My son did not tell me about this” the patient exclaimed repeatedly. The dentist tried to comfort her and asked if she would like to speak to her son to confirm that he had scheduled the appointment. The dentist used her own cell-phone and put the son on speaker to comfort his mother. The patient, relieved by hearing her son’s voice asked him repeatedly to “stay” with her. The dentist placed the phone near the patient’s bed and assured the patient that her son could stay with them for the entire session. Hearing the voice of her son comforted this person and allowed the dentist to perform the treatment (dental filling).	

^a^Note: some dentists in this study were not in favor of medicating patients and preferred them to remain aware of their presence and what they were doing. When attempts to gain patient’s collaboration fail, they preferred to stop and to reschedule the appointment for a different day.

In terms of interactions within the dental team, the dentists communicated updates with their administrative staff in different ways. One dentist would send an electronic image of the patient’s chart (in LTCFs, paper charts were retained in the patient’s medical file) via “Dropbox” to his administrative staff. Another dentist used a laptop to complete files after every appointment while the remotely-based secretary had online access to the same software. Conversely, in the clinic where the secretary accompanied the team, the dentist would dictate the updates to the secretary who completed patients’ electronic files during appointments.

#### Logistics and treatment

##### Scheduling and planning visits

All administrative tasks (such as scheduling and billing) were performed on a dental practice management software, just like in conventional clinics. This said, these tasks were sometimes more complex and required more coordination and follow-up given the multiple levels of communication required; and the need to consider the patients’ locations in order to geographically pool visits, reduce traveling time, and maximize efficiency.

For example, a clinic manager described how they sometimes had to wait for family members/legal representatives to give consent in order to schedule visits. Occasionally, seeking those approvals took longer than anticipated. Once consent was obtained, for patients residing in LTCFs, the clinic’s manager had to contact the LTCF staff to inform them of the planned visit and to finalize scheduling. Then, a final confirmation call was usually done the day before the visits.

An important consideration while scheduling appointments, according to several members of the dental team, was the pooling of visits by neighborhood or town, on any given day. A clinic manager highlighted how this was not an easy task, especially in the beginning of joining the mobile team. However, with experience, this team member explained, planning weekly and daily schedules became easier by learning to estimate length of visits and distance between locations, as well as considering other factors such rush-hour traffic. In the three clinics, the schedules were planned weeks in advance. The time allocated per visit varied between the three clinics. In LTCFs the duration ranged from 30minutes (Clinic C) to 60 minutes (Clinic A) per appointment. Home-visits, on the other hand, may require longer appointments of 1.5-2 hours (Clinic B) due to varying traveling and setup durations.

Subsequently, another logistics challenge that arose for mobile clinics was emergency appointments or urgent follow-ups. With carefully planned, area-specific schedules, the dental teams found it often difficult to “fit in” new appointments in random locations. One potential solution described by the dentists is to reserve a half or full day for emergency visits. However, given the high demand and low supply of domiciliary dentistry, they remained unsure about the most ideal approach and had not implemented anything particular in their clinics.

In order to increase availability of domiciliary dentistry, one dentist believed in the adoption of what they coined “proximity dentistry”. In this model, existing traditional clinics would offer a mix of fixed and mobile services within a one Kilometer radius of the clinic.

##### Accessing the domiciles

Weather conditions and/or accessibility of domiciles sometimes complicated the process of domiciliary dentistry. Quebec’s winter weather conditions are sometimes harsh. Snow storms lead to reduced visibility for commuters and piled snow or icy sidewalks become a hazard while moving dental units and equipment. Additionally, the type of domicile may further complicate access due to multiple stairs, lack of elevators, distance from parking to entrance, etc.

Both LTCFs and private homes had their unique advantages and disadvantages in terms of access. LTCFs typically offered: 1) designated parking spaces; 2) multiple patients in one location; 3) easy access to medical charts with detailed medical history and medication information; and 4) rooms equipped with electric beds and transfer-lifts (operated by the nurses), which facilitated positioning patients for treatment.

The disadvantages of LTCFs were mainly related to strict access protocols or dealing with staff. For example, sometimes the dental team faced delays because the reception were not informed of the anticipated visit, or because they needed to ask for directions to the patient’s room. Sometimes the staff seemed unhelpful or would just refer the team to another member of the staff. The dentists sympathized with the staff and pointed that they were probably “overworked” due to the centers being understaffed.

On the other hand, in private homes or apartments, accessibility also varied depending on: 1) physical accessibility of the home (parking issues, stairs, etc.); and 2) adaptability of the dental team to the different social contexts of home visits. Dentists with strong social communication skills could more readily navigate different norms and cultures inside patients’ homes. For some dentists this was considered an overwhelming and distracting task. Others, however, highlighted the value of such interaction for themselves and for the patients:

*I am someone who loves to have contact with people, so my first appointment, before the pandemic, was to take a coffee, talk with my patient, to really understand the profile of my patient. It was my approach… They* [the patients and their families] *are usually tired and stressed, we are there for them… a mobile dentist is way more than just that. So, if I can provide social support, moral support, and indirectly, we can create a network with the nurse (and) the CLSCs* [French acronym for the local community service centers].(Dentist, clinic B)

*It is a gift to go to homes. We enter their intimate space. They trust us and include us in their social life*. (Dental assistant, clinic C)

*Patients need them* [home visits], *there is a lot of demand. They are very appreciative, and the people are very nice… I got two cans of spaghetti last week!* (Dentist, clinic A)

##### Set up and treatment

After entering a domicile, the dental teams began the process of positioning the patient, stationing the mobile dental unit, and then preparing the patient for examination and treatment. At LTCFs, the patients were usually positioned in their hospital beds. Electric hospital beds were convenient for the patients and ergonomically well suited for the dental team. In homes, patients were usually set up in their own wheelchairs; beds (conventional or electric); or in reclining arm chairs (such as the popular “Lazyboy”). Sometimes the dentist requested and assisted the patient to transfer to a more convenient spot (for example from wheelchair to bed).

To station the dental unit, the dental team located the closest power outlet and, depending on the nature of the visit, looked for the nearest sink (to rinse a denture, for example).

The last step in the set-up process was to prepare the patient by brushing the teeth and/or gums to remove any residue and facilitate examination. This step was considered essential as patients typically had poor oral hygiene practices. One dentist used a high-concentration chlorhexidine toothpaste as a prophylactic first-step for every patient. Another dentist only used a wet brush to clean the oral cavity and provided a high-concentration fluoride gel to patients on a need-basis.

The range of treatments performed by the dentists included: cleaning, dental fillings, abscess drainage, extractions, and some denture repair. According to the dentists, all dental treatments could potentially be performed by a mobile dentist, including endodontics and implants. However, given the prohibited use of mobile x-ray devices in the province of Quebec, they were sometimes reluctant to perform certain procedures. The main characteristics of the dental treatments were: 1) the focus on prevention and preservation as most patients could not tolerate lengthy restorative procedures; and 2) the use of dental materials that are easy to manipulate and have favorable properties such as Silver Diamine Fluoride (SDF) or glass ionomer. One dentist described SDF as an ideal material for arresting and preventing caries, that is also easy to apply and did not require any specialized equipment.

At the end of each session, used instruments were placed in a covered container to be returned t the storage facility for sterilization, at the end of the day.

##### Financial aspects

The dentists acknowledged concerns among dental professionals over the financial viability of domiciliary dentistry. However, they emphasized that they were able to build a profitable service model. They argued that although financial gain may be slow at the beginning, mobile dentists who were just starting needed to give it time. One dentist explained that it took a lot of adjustment when starting: “it [was] very difficult to move around [LTCFs] at first…when I first began with my assistant, I remember it would take us half an hour just to get from the parking to the room!”. “At the beginning it’s slow, but now it’s very good!” said another dentist referring to financial returns after less than two years in mobile practice. The three clinics set their fees in accordance with the provincial syndicate fee-guide (just like conventional clinics). Additionally, two of the clinics charged a fixed displacement fee per patient ($100 and $110, respectively). According to a dentist who did not charge the extra fee, they compensated travel costs by pooling patients in LTCFs to maximize the number of appointments per location.

One dentist believed that the prevailing concerns about poor remuneration for mobile dental services among dentists are rooted in the approach dental schools take in training dental professionals and confirm the persistent inequities in access to oral healthcare:

*Even in a conventional clinic, treating geriatric patients or patients with disabilities is not money making! it requires more time, more appointments… but that’s not what we were taught in school. At school we learn to do crowns, fill cavities, but we don’t learn to treat the human!... I don’t have a choice* [regarding the displacement fee]*, if I don’t do it, it* [the service] *doesn’t pay back… A lot of people don’t take my services because it’s expensive, but what I charge is the least I can charge for my company to be running.*

## Discussion

We studied three cases of domiciliary dentistry in the province of Quebec, Canada. We described the main features of their set up including operationalization, feasibility, advantages, and limitations. We also examined the service features including attitudes, interactions, and financial aspects. Our case study analysis and the following discussion points and recommendations could be transferred or adapted to other contexts of oral healthcare delivery.

### Feasibility of domiciliary dentistry

In this study, the dentists chose to invest in commercially available or customized portable dental units. The market offers several types of portable dental units and a quick internet-search yields multiple options at various prices ranges. Notably, despite the well-equipped dental units in the three clinics, the range of feasible procedures was limited by the prohibition of portable X-ray units in domiciliary settings in Quebec.

Dentists who are reluctant to invest in a portable unit may consider alternative lower-cost options such as rechargeable (battery-operated) hand pieces and portable suction [12, 13]. Alternatively, dentists may want to explore this type of practice by starting with a simple selection of instruments to perform basic dental care before eventually building their mobile practice and adding more sophisticated equipment [5, 14].

In terms of logistics, a common challenge for our clinicians was scheduling emergency appointments and urgent follow-ups especially when the dentists had their pre-planned schedule in areas far from the emergency request. This challenge could also be attributed to the high demand for domiciliary dentistry and low number of mobile dentists offering this service [15]. One dentist envisioned the solution of “proximity dentistry”, where traditional clinics would offer domiciliary services within a small radius. Subsequently, when such a model is reproduced in different neighborhoods and locations, it would help with the issue of travel time for both dentists and patients, as well as increase the reach of mobile dentistry to those in need.

### Profitability of domiciliary dentistry

We found domiciliary dentistry to be profitable for the dental professionals in several ways. From an operational perspective, domiciliary dentistry offers a flexible work environment [5], a change of scenery [12, 13], and may allow for more work-life balance. Secondly, from a service perspective, providing access to care to those unable to seek it themselves can be highly rewarding for dental professionals [14, 16]. Domiciliary dentistry is a model of practice that reflects person-centered care, equity, and inclusion; which are values that healthcare providers aspire to. Moreover, we found that domiciliary dentistry involves rich human experiences that are meaningful and potentially transformative.

From a financial perspective, despite the commonly cited concerns about poor remuneration [6, 12, 17]; The clinics’ owners in this study confirmed the contrary; stating that this model can eventually be profitable. They also used multiple strategies to augment income such as: 1) charging a displacement fee; 2) focusing on LTFCs where multiple patients can be seen in one visit; and 3) Increasing efficiency during and between visits by increasing the size of the dental team or purchasing more dental units for LTCFs visits. The literature also points towards concerns about the costs of setting up and buying the necessary equipment [5, 18], however, this was not a concern among the dentists in this study. They did not hesitate to invest in portable dental units because they believed in the potential and need for such a service, hence, justifying the initial costs.

Additionally, treating patients with various physical or cognititve disabilities indeed requires longer appointments and the time-in-transit between patients may sometimes be considered a loss of income-opportunities. This is particularly amplified when a dentist practicing in a fixed clinic attempts to add-on domiciliary services. In this case, the running costs of a fixed clinic would be difficult to compensate for. One solution in the case of mixed-models of practice is to hire associates who would replace the mobile dentist in the fixed clinic. Nonetheless, our mobile dentists highlighted the need for a fundamental shift from prevailing treatment-focused attitudes within the dental profession towards a more holistic and person-centered approach in order to fight persisting inequities [13].

This attitudinal shift is important because seniors and people with disabilities may have financial insecurities due to loss of income and subsequently, loss of dental coverage from employment insurance. This situation, coupled with the lack of government coverage for dental services for adults in countries like Canada, further aggregates the financial barriers rendering dental services (including domiciliary dentistry) unattainable for a growing segment of the population.

### Value of domiciliary dentistry

Research has shown that dependent elderly and people with disabilities feel less anxious and become more involved in their dental care when it’s provided in a familiar environment [14, 19]. Domiciliary services also reduce the burden on caregivers who would otherwise need to carefully move the patients, bring them to ta clinic, and manage unpredictable accessibility issues. Both patients and caregivers expressed immense gratitude and appreciation for the dental teams’ kindness, patience, and skills. Ultimately, as one dentist explained, increasing all types of domiciliary services (including oral healthcare) promotes the autonomy of seniors allowing them to stay in their own homes as long as possible.

### So, What can be done to promote domiciliary dental services?

At the personal level, dentists who are interested in providing services for dependent elderly and people with disabilities could benefit from continuing education courses that would familiarize them with the specifics of caring for geriatric patients or patients with particular conditions such as Alzheimer’s, for example. At a professional level, authorities could develop policies that support the practice of domiciliary dentistry and, in contexts such as Quebec, Canada, permit the use of portable X-ray units to improve the scope of domiciliary dental procedures. Furthermore, on a structural level, dental schools could play a major part in shaping future dental professionals’ attitudes towards alternative models of dental practice through targeted training, community outreach programs, and interdisciplinary collaborations with other healthcare professionals. Finally, national healthcare systems could also play a significant role by developing and promoting oral healthcare strategies that would increase access to care for all member of society such as mandating oral healthcare services in LTCFs [20].

## Conclusion

Domiciliary dentistry is a feasible and potentially profitable alternative model of dental practice in Quebec, Canada. The three clinics we studied varied in terms of the number of years in mobile practice and the structure and size of their teams, however, they also shared similarities in their physical and service features. All three clinics exclusively offered domiciliary dental services in homes and/or LTCFs. Their physical set up comprised of mobile dental units, a storage/sterilization location, and a vehicle for transportation. They faced similar logistic challenges such as scheduling urgent appointments, interacting with patients, and the limiting ban on portable x-ray units. However, they were all able to mitigate these challenges to respond to the high demand for domiciliary dental services. Another common feature was the personality traits of the dentists and their team members who were remarkably patient and adaptable.

This study contributes to building evidence-based models of domiciliary dentistry. More research from different contexts can continue to examine alternative models of oral healthcare delivery. Also, given the calls for curricular shifts in undergraduate dental programs towards more patient and society centered dentistry; future studies could assess the impact of educational intervention on new graduates’ attitudes towards domiciliary dentistry.

## Supplementary Information

**Additional file 1.**

## Data Availability

The datasets generated and/or analysed during the current study are not publicly available due to potential to compromise anonymity of participants but are available from the corresponding author on reasonable request.
