# Supplementary material for: Domiciliary dentistry clinics: a multiple case study in the province of Quebec, Canada
Source: BMC Health Serv Res. 2021 Sep 15;21:972. doi: 10.1186/s12913-021-06788-4 (PMC8442809; doi:10.1186/s12913-021-06788-4)
Supplement: Supplementary file 1 — Additional file 1. [file 12913_2021_6788_MOESM1_ESM.pdf]

Interview guide (1)

Dental team

Professional background:

- ➢ Invite participant to introduce him/herself and describe their professional background.

  Examples of questions:

- What is your role/responsibility in this clinic?
- How did you become involved in mobile dentistry (motivation)?
  How long have you been working in mobile dentistry?
- Have you worked (or currently working) in other clinics?

Mobile dentistry setting

- ➢ Invite participant to describe the history of the mobile clinic

  Example question: How did you set up the mobile clinic? How did you recruit staff? (for owner)

- ➢ Invite participants to describe the physical set up of their mobile clinic

  Examples of questions:

- What equipment do you use in this mobile clinic? What is the cost?
- How do you store and transport equipment?
- What are the challenges with mobile equipment? How do you overcome those challenges?
- What are the advantages of mobile dentistry?
- What are the challenges of mobile dentistry? How do you overcome those challenges?

Provision of domiciliary services

Example question: Describe a typical work day for you…

Examples of follow-up questions:

- Who do you offer domiciliary services to?
- Where do you offer your services? What types of domiciles do you visit?
- What is the range of treatments you offer?
- How many patients do you see?
- How about scheduling and appointment planning?
- What is the cost of domiciliary dental services? And how do you determine the rates?

Experience with patients/caregivers

- Invite participant to describe their interaction with patients/caregivers

Examples of questions:

- How would you describe your experience with patients/caregivers in general?
- Could you share your positive experiences (satisfactions)?
- What types of challenges do you encounter when interacting with patients/caregivers? How do you overcome them?
- Are there different challenges depending on the condition (health, type of disability, age, etc.) of patients?

Supply of domiciliary dental services

- Invite participant to comment on the lack of domiciliary dental services and the commonly reported negative perceptions of domiciliary dentistry (e.g. demanding patient population, low profitability)

Examples of question:

- Why is there a reluctance among dentists to practice domiciliary dentistry?
- How can we increase the uptake of domiciliary dentistry among practicing and future dentists?

Final comments

- Invite the participant to share any comments or feedback they wish to add

Interview guide (2)
Patients/caregivers

General and oral health status

- Invite participant to introduce him/herself and describe their general health.

  Examples of questions:

- Tell me a little bit about yourself, your age, your health status (probe for details)
- Tell me about your oral health. How would you describe it?
- Considering your [medical-physical] condition, what is the place of oral health in your life?

Access to oral healthcare

- Invite the participant to describe their current and past dental consultations

  Examples of questions

- How long have you been seeing the mobile dentist?
- Why did you seek domiciliary services?
- Describe your dental consultations before starting with the mobile dentist, what were the challenges?
- Do you have dental insurance?

Experience of domiciliary dental services

- Invite the participant to describe the last appointment they had with the mobile dentist

Examples of questions:

- When was the last appointment?
- What was the reason for the consultation?
- How did you arrange the appointment?
- What did the dentist do?
- How did it go?

Attitudes towards domiciliary dentistry

- Invite the participants to describe their overall experience of domiciliary dental care

  Example question: Tell me about your experience with the mobile dentist/dental team. What do you like/dislike?

Final comments

- Invite the participant to share any comments or feedback they wish to add
